# S04-3 The effectiveness of a coordinated action between the healthcare sector and local sports clubs to promote physical activity: an intervention trial

**DOI:** 10.1093/eurpub/ckac093.019

**Published:** 2022-08-29

**Authors:** Sylvia Titze, Wolfgang Ruf, Christian Lackinger, Lena Großschädl, Albert Strehn

**Affiliations:** 1 University of Graz, Institute of Human Movement Science, Graz, Austria; 2 Institute of Sport Science, German University of Health and Sport, Berlin, Germany; 3 Kuratorium Wiener Pensionisten-Häuser, Vienna, Austria; 4 Head Office Styria, Social Security Service for Entrepreneurs, Graz, Austria; 5 Head Office Burgenland, Social Security Service for Entrepreneurs, Eisenstadt, Austria

**Keywords:** Exercise, Health sector, Sport sector, Physical activity counselling

## Abstract

**Background:**

Joining forces through collaboration between the health- and sports-sector led to the project Jackpot.fit, which aimed at increasing physical activity (PA) among inactive adults in Styria (a state of Austria). The main objective was to assess the short- and long-term effectiveness of a mixed PA intervention, consisting of counselling in a health care setting combined with a standardised sports club programme, on PA in an adult population.

**Methods:**

In a quasi-experimental study with two follow-up time points (4 and 12 months after baseline), participants from eight regions were allocated to the intervention group (IG) and participants from three other regions to the comparison group (CG).

Half of the study participants were women and the mean participants' age was 53 (*SD* ± 6) years. During the health resort stay, the IG received PA counselling and coupons for 12 complimentary, standardised training sessions in a sports club near the participants' home. Participants of the CG received identical PA counselling, but written material only. PA was measured with an accelerometer (GENEActive). Linear mixed-effects models were applied to examine changes of PA within and between groups over time.

**Results:**

From 217 participants (IG = 167; CG = 50) at least one follow-up measurement was available. IG data showed a significant increase in moderate-intensity PA from baseline (101 min/week) to 4 months (+58 min; 95% CI = 36 to 80) and 12 months (+24 min; 95% CI = 2 to 46) whereas there was no significant change in CG. Between-group comparisons revealed a significant difference after 4 but not after 12 months.

**Conclusions:**

The study confirms the feasibility of coordinated actions between health care institutions and sport clubs to recruit people into a standardized PA programme. In addition, those who participated in the sports club programme increased their PA in a long term.

